# Metabolic characterization of isocitrate dehydrogenase (IDH) mutant and IDH wildtype gliomaspheres uncovers cell type-specific vulnerabilities

**DOI:** 10.1186/s40170-018-0177-4

**Published:** 2018-04-17

**Authors:** Matthew Garrett, Jantzen Sperry, Daniel Braas, Weihong Yan, Thuc M. Le, Jack Mottahedeh, Kirsten Ludwig, Ascia Eskin, Yue Qin, Rachelle Levy, Joshua J. Breunig, Frank Pajonk, Thomas G. Graeber, Caius G. Radu, Heather Christofk, Robert M. Prins, Albert Lai, Linda M. Liau, Giovanni Coppola, Harley I. Kornblum

**Affiliations:** 1 Department of Neurosurgery, and the Interdepartmental Program in the Neurosciences, University of California, Los Angeles, CA 90095 USA; 2 Department of Molecular and Medical Pharmacology, David Geffen School of Medicine, UCLA, Room 379 Neuroscience Research Building, 635 Charles E. Young Dr. South, Los Angeles, CA 90095 USA; 3 UCLA Metabolomics Center, UCLA, Los Angeles, USA; 4 Department of Chemistry and Biochemistry, UCLA, Los Angeles, USA; 5 Ahmanson Translational Imaging Division, UCLA, Los Angeles, USA; 6 Department of Psychiatry and Biobehavioral Sciences and Semel Institute for Neuroscience & Human Behavior, UCLA, Room 379 Neuroscience Research Building, 635 Charles E. Young Dr. South, Los Angeles, CA 90095 USA; 7 Department of Human Genetics, UCLA, Los Angeles, USA; 8 Board of Governors Regenerative Medicine Institute, Cedars-Sinai Medical Center, Los Angeles, CA USA; 9 Samuel Oschin Comprehensive Cancer Center, Cedars-Sinai Medical Center, Los Angeles, CA USA; 10 Department of Biomedical Sciences, Cedars-Sinai Medical Center, Los Angeles, CA USA; 11 Department of Radiation Oncology, David Geffen School of Medicine at UCLA, Los Angeles, USA; 12 Jonsson Comprehensive Cancer Center, UCLA, Room 379 Neuroscience Research Building, 635 Charles E. Young Dr. South, Los Angeles, CA 90095 USA; 13 Department of Neurology, UCLA, Los Angeles, USA; 14 Eli and Edythe Broad Center of Regenerative Medicine and Stem Cell Research, UCLA, Room 379 Neuroscience Research Building, 635 Charles E. Young Dr. South, Los Angeles, CA 90095 USA

**Keywords:** 2-hydroxyglutarate, Metabolism, Nucleotide, Radiation, Glioma

## Abstract

**Background:**

There is considerable interest in defining the metabolic abnormalities of IDH mutant tumors to exploit for therapy. While most studies have attempted to discern function by using cell lines transduced with exogenous IDH mutant enzyme, in this study, we perform unbiased metabolomics to discover metabolic differences between a cohort of patient-derived IDH1 mutant and IDH wildtype gliomaspheres.

**Methods:**

Using both our own microarray and the TCGA datasets, we performed KEGG analysis to define pathways differentially enriched in IDH1 mutant and IDH wildtype cells and tumors. Liquid chromatography coupled to mass spectrometry analysis with labeled glucose and deoxycytidine tracers was used to determine differences in overall cellular metabolism and nucleotide synthesis. Radiation-induced DNA damage and repair capacity was assessed using a comet assay. Differences between endogenous IDH1 mutant metabolism and that of IDH wildtype cells transduced with the IDH1 (R132H) mutation were also investigated.

**Results:**

Our KEGG analysis revealed that IDH wildtype cells were enriched for pathways involved in de novo nucleotide synthesis, while IDH1 mutant cells were enriched for pathways involved in DNA repair. LC-MS analysis with fully labeled ^13^C-glucose revealed distinct labeling patterns between IDH1 mutant and wildtype cells. Additional LC-MS tracing experiments confirmed increased de novo nucleotide synthesis in IDH wildtype cells relative to IDH1 mutant cells. Endogenous IDH1 mutant cultures incurred less DNA damage than IDH wildtype cultures and sustained better overall growth following X-ray radiation. Overexpression of mutant IDH1 in a wildtype line did not reproduce the range of metabolic differences observed in lines expressing endogenous mutations, but resulted in depletion of glutamine and TCA cycle intermediates, an increase in DNA damage following radiation, and a rise in intracellular ROS.

**Conclusions:**

These results demonstrate that IDH1 mutant and IDH wildtype cells are easily distinguishable metabolically by analyzing expression profiles and glucose consumption. Our results also highlight important differences in nucleotide synthesis utilization and DNA repair capacity that could be exploited for therapy. Altogether, this study demonstrates that IDH1 mutant gliomas are a distinct subclass of glioma with a less malignant, but also therapy-resistant, metabolic profile that will likely require distinct modes of therapy.

**Electronic supplementary material:**

The online version of this article (10.1186/s40170-018-0177-4) contains supplementary material, which is available to authorized users.

## Background

Alteration in cellular metabolism is a key pathway to the development of cancer. Most oncogenes and tumor suppressors influence cellular metabolism, and there are examples of mutations in metabolic genes that become tumorigenic [1]. Homologous mutations in the metabolic enzymes isocitrate dehydrogenase 1 and 2 (*IDH1* and *IDH2*) are found in acute myelogenous leukemia, colon cancer and glioma [2]. In glioma, these mutations generally co-occur with either 1p/19q co-deletion or with mutations in *TP53*, the former with more oligodendrocytic characteristics and the latter with more astrocytic characteristics [3]. In contrast to metabolic mutations that involve a loss of function, IDH mutations were found to bestow a new enzymatic function of reducing alpha-ketoglutarate (a-KG) to 2-hydroxyglutarate (2-HG) [4]. In the presence of IDH mutations, the 2-HG molecule, normally found at low levels, can increase to millimolar amounts. Understandably, there has been considerable interest in what role this potential “oncometabolite” might have on cells. Given the structural similarity of the 2-HG molecule to a-KG, it was suspected that 2-HG may be a competitive inhibitor that blocked access to a-KG-dependent enzymes that regulate cell epigenetics [5, 6].

There has been considerable interest in defining what effects IDH mutations have on glioma cell biology, and what the discovery of an IDH mutation can tell us about a specific glioma. These questions are important for two reasons. The first reason comes from the rationale that if the IDH mutant enzyme changes the metabolic state of the cell, this may render the cell more or less vulnerable to certain types of therapy. For example, it has been reported that the IDH1mutation makes cells more vulnerable to radiation [7] or NAD+ depletion [8]. This issue has become even more clinically relevant with the discovery that the presence of IDH mutations can be diagnosed via imaging even prior to surgery [9]. The second reason comes from the observation that glioblastoma patients with IDH mutant tumors have prolonged survival compared to patients with IDH wildtype tumors and that there are now pharmacological inhibitors of the IDH mutant enzymes available that block 2-HG formation [10]. If it is the case that the IDH mutation is actually a metabolic burden to the cell, then use of these inhibitors may actually aid the tumor cell and accelerate growth. Studies using mutant IDH inhibitors in in vivo xenograft models have led to mixed results with one showing slowed growth [11] and another showing accelerated growth [8].

Attempts to focus on isolating metabolic differences between IDH mutant and IDH wildtype glioblastomas have historically suffered from an unproven assumption that the metabolic differences between IDH mutant and IDH wildtype tumors can be largely attributed to the presence or absence of the IDH mutation itself [12, 13]. However, more recent evidence suggests that IDH mutation may be one of the initial mutations to occur in those gliomas [14, 15]. Large-scale bioinformatics analyses of mutational, expression and epigenetic datasets reveal that IDH mutant and IDH wildtype tumors are different on a very fundamental level [16] and may have different cells of origin, and different paths of tumorigenesis. Unfortunately, attempts to study cells derived from endogenous IDH mutant tumors have been hampered by the difficulty involved in establishing and maintaining IDH mutant glioma cells in culture.

To address these issues, we have performed a metabolic analysis on a cohort of patient-derived IDH1 mutant and IDH wildtype tumor cells to determine differences between these groups that may potentially be exploitable for therapy. We demonstrate that when compared to IDH wildtype glioma cells and tumors, IDH1 mutant cells and tumors are enriched for gene sets associated with DNA repair, while wildtype cells have greater expression of gene sets associated with nucleotide biosynthesis. IDH1 mutant cells have metabolic profiles that are distinct from those of IDH wildtype cells and our findings indicate that at least some of these differences are not corrected by overexpression of mutant IDH1 in IDH wildtype cells. Functional studies surprisingly demonstrate that IDH1 mutant cells are better able to recover from radiation treatment and less prone to the effects of inhibition of de novo nucleotide biosynthesis. These findings indicate that IDH mutant and wildtype tumors may be responsive to different metabolically directed therapies and that the previously held views that IDH mutant tumors are highly radiosensitive may not be correct for all subsets of IDH mutant tumors and needs further exploration.

## Methods

### Collection of in vitro cultures

Samples were collected under institutional review board-approved protocols and graded by neuropathologists. We previously reported on gene expression analysis in these samples in all except one of the IDH mutants [17]. IDH wildtype samples were all from GBM. Of the seven IDH1 mutant cultures, five were from GBM, one from a grade III oligoastrocytoma and one from a grade II oligodendroglioma. Cultures were prepared as described previously [17]. Briefly, on the day of resection, samples were digested with papain. Acellular debris was removed and remaining cells were incubated in gliomasphere media (DMEM/F12 supplemented with B27, penicillin/ampicillin, heparin, EGF and bFGF) for several days until spheres began to form. Frozen stocks were made at passage 5 to maintain cells at low passage. One IDH1 mutant line, BT142, was obtained through ATCC [18, 19]. Relevant information pertaining to the cultures used for in vitro experiments is summarized in Additional file 1, which provides the characteristics of the patients and the tumor as reported by neuropathologists as well as data from comparative genomic hybridization and whole exome sequencing in the samples for which it was available. The majority of experiments were performed using the same 3 IDH WT lines (HK157, HK301, and HK308) and 3 IDH1 mutant lines (HK213, HK252, and HK322). Prior to the completion of data collection, the HK322 culture was lost. Therefore, two additional IDH1 mutant lines (BT142 and HK211) were included for the radiation experiments.

### Whole exome sequencing and mutation analysis

Genomic DNA was extracted and fragmented by sonication using the Covaris acoustic disruptor (model E210, Covaris Inc.) to achieve an average fragment size of 200 base pairs. Two hundred nanograms of DNA from each sample were used for the construction of DNA libraries using Kapa Hyper DNA library prep kits and matched dual index adapters (Integrated DNA Technologies). Exome capture was performed using the Nimblegen SeqCap EZ Exome enrichment kit. Sequencing was performed on an Illumina HiSeq 4000 following the manufacturer’s instructions. 65–70 million 120-base, paired-end reads were obtained per sample on average, with a 50× average depth of coverage within the targeted exome. Raw image files were processed with the Illumina CASAVA 1.8 software (Illumina, San Diego, CA).

Whole exome sequencing was analyzed using the Genome Analysis Toolkit (GATK) [20] pipeline. Briefly, short reads were aligned using the Burrows-Wheeler Aligner (BWA) [21]. SAMtools was used to convert between SAM and BAM file format, and Picard tools to sort alignments and mark duplicates. Variants were called using the GATK HaplotypeCaller function with the following parameters: variant index parameter 128,000; variant index type LINEAR; nda; maxAltAlleles 4; ERC GVCF; contamination 0.02. ANNOVAR [22] was used to annotate variants.

### Copy number variation (CNV) analysis

DNA libraries were hybridized onto Affymetrix CytoScanHD arrays. We first processed the raw intensity CEL files using the R package affy2sv [23] to generate B-allele frequency (BAF) and log R ratio (LRR) values. Secondly, we used the PennCNV software (parameters: exome HMM model with gcmodel adjustment, filtered by 10 SNPs minimum 10 and with region length longer than 50 k) [24], the R package GenoCN (parameters: cnv-only snpInfo$PFB > 1; outputSNP 1; outputSeg TRUE) [25], and the R package Rawcopy (default parameters) [26] for CNV inference. CNV calls were obtained by integrating the output from all callers.

### Gene set enrichment analysis

RNA was purified from 59 patient-derived gliomasphere cultures and hybridized to Affymetrix U133 Plus 2.0 arrays. For KEGG-based analysis, we collapsed gene expression probes based on enzyme activity (Enzyme Commission numbers [EC]) rather than on gene identity to avoid unequal representation of equivalent enzymatic function within pathways, thus emphasizing potential flux through the network. The metric used for gene ranking was the signal to noise ratio (SNR) between the IDH1 mutant and IDH1WT samples. The metric was calculated for all candidate probesets of each gene or enzymatic activity and the probeset with maximum absolute metric value was retained. Probeset annotation was based on UniGene build #201, and UniGene identifiers were mapped to each EC using the gene names provided by KEGG. Pathways with fewer than three or greater than 500 nodes represented by the data were excluded from the analysis. This resulted in 167 KEGG modules in the TCGA dataset and 186 modules in the gliomasphere data set.

### Glucose/glutamine uptake

200,000 cells were plated in 3 ml of gliomasphere media and allowed to grow for 24 h. The cells were spun down and counted, and glucose and glutamine levels in the used media were measured using a NOVA Bioanalyzer and compared to blank control [27–29]. To evaluate the effects of IDH1 mutant inhibition on glucose/glutamine uptake, 5 μM of c227 was added to the culture 24 h before being harvested.

### Cell proliferation studies (CFSE)

Cell proliferation of endogenous IDH1 mutant and IDH wildtype cultures was assessed using CFDA SE (CFSE; carboxyfluoresceindiacetate, succinimidyl ester) cell tracer kit (Invitrogen) as described previously [30]. Briefly, spheres were dissociated with Accumax (Innovative Cell Technologies), stained with CFSE according to manufacturer suggestions, and grown for 5 days under normal gliomasphere conditions at a density of 100,000 cells/mL. Preparation for FACS analysis was performed according to manufacturer guidelines using 4% paraformaldehyde as a fixative. Following FACS acquisition, Proliferation Wizard Basic Model was used to assess proliferative populations within each sample and to generate average division times.

### Liquid chromatography–mass spectrometry (LC-MS)

Cells were cultured for 24 h and rinsed with PBS, and either unlabeled media, 50% ^13^C-glucose labeled media, or 50% ^13^C-glutamine labeled media was added. After 24 h, cells were rinsed with ice-cold 150 mM NH_4_AcO (pH 7.3), followed by addition of 400 μl cold methanol and 400 μl cold water. Cells were transferred to an Eppendorf tube, and 10 nmol norvaline (Sigma-Aldrich, N7502) as well as 400 μl chloroform was added to each sample. For the metabolite extraction, samples were vortexed for 5 min on ice and spun down, and the aqueous layer was transferred into a glass vial and dried. Metabolites were resuspended in 70% ACN and 5 μl loaded onto a Phenomenex Luna 3u NH_2_ 100A (150 × 2.0 mm) column. The chromatographic separation was performed on an UltiMate 3000 RSLC (Thermo Scientific) with mobile phases A (5 mM NH_4_AcO pH 9.9) and B (ACN) and a flow rate of 300 μl/min. The gradient ran from 15% A to 95% A over 18 min, 9 min isocratic at 95% A, and re-equilibration for 7 min. Metabolite detection was achieved with a Thermo Scientific Q Exactive mass spectrometer run in polarity switching mode (+ 3.0 kV/− 2.25 kV). TraceFinder 3.3 (Thermo Scientific) was used to quantify metabolites as the area under the curve using retention time and accurate mass measurements (< 3 ppm). Relative amounts of metabolites were calculated by summing up all isotopologues of a given metabolite and normalized to the internal standard and cell number. Clustering analysis was done in R.

### Metabolic tracing using combined liquid chromatography and tandem mass spectrometry (LC-MS/MS)

Cell lines were grown in gliomasphere media supplemented with fully labeled ^13^C-glucose and ^13^C9,^15^N3-dC. The cells were allowed to grow for 48 h at which point they were harvested and lysed. Genomic DNA was extracted using the Quick-gDNA MiniPrep kit (Zymo Research, D3021) and hydrolyzed to nucleosides using the DNA Degradase Plus kit (Zymo Research, E2021), following manufacturer-supplied instructions. In the final step of DNA extraction, 50 μL of water was used to elute the DNA into 1.5 mL Eppendorf tubes. A nuclease solution (5 μL; 10× buffer/DNA Degradase Plus™/water, 2.5/1/1.5, *v*/*v*/*v*) was added to 20 μL of the eluted genomic DNA in an HPLC injector vial. The samples were then incubated overnight at 37 °C. Samples (20 μL) were injected onto a porous graphitic carbon column (Thermo Fisher Scientific Hypercarb, 100 × 2.1 mm, 5 μm particle size) equilibrated in solvent A (water/acetonitrile/formic acid, 95/5/0.2, *v*/*v*/*v*) and eluted (200 μL/min) with an increasing concentration of solvent B (acetonitrile/water/formic acid, 90/10/0.2, *v*/*v*/*v*) using min/%B/flow rates (μL/min) as follows: 0/0/200, 5/0/200, 10/15/200, 20/15/200, 21/40/200, 25/50/200, 26/100/700, 30/100/700, 31/0/700, 34/0/700, 35/0/200. The effluent from the column was directed to the Agilent Jet Stream ion source connected to the triple quadrupole mass spectrometer (Agilent 6460) operating in the multiple reaction monitoring mode using previously optimized settings. The peak areas for each nucleosides and nucleotides (precursor→fragment ion transitions) at predetermined retention times were recorded using the software supplied by the instrument manufacturer (Agilent MassHunter). To evaluate the effects of IDH1 mutant inhibition on glucose glutamine labeling, 5 μM of c227 was initially added to the culture and then re-added following PBS wash.

### Deoxythymidine (dT) treatment

To evaluate the effects of nucleotide synthesis inhibition, a group of cells were grown in gliomasphere media and subjected to 1 mM dT treatment and allowed to grow for 4 days. At this point the cells were harvested, stained with DAPI, and subjected to flow cytometry analysis to determine cell cycle distribution.

### Radiation

To determine the extent of DNA damage following radiation, gliomaspheres were dissociated, plated at 1 × 10^5^ cells/mL, and subjected to a 10 Gy dose of radiation using an X-ray irradiator (Gulmay Medical, Atlanta, GA) (5.519 Gy/min; 250 kV; a 4-mm Be, a 3-mm Al, and a 1.5-mm Cu filter). Neutral-buffered Oxiselect Comet Assay Kit (Cell Biolabs) was used to assess DNA damage according to manufacturer’s instructions. For the c227 comet experiments presented in Additional file 2, IDH1 mutant cells were treated with 5 μM c227 or control for 48 h prior to irradiation (10 Gy) and comet analysis as described above. Radiation-induced apoptosis was measured at 4 days after treatment using TUNEL staining. To determine effects of growth following radiation, cell lines were plated at 2 × 10^5^ cells in 3 ml of gliomasphere media. They were then subjected to the indicated doses of radiation (0, 2, 6, and 10 Gy) and allowed to recover for a minimum of 4 days. When the control group of each cell line was ready to be passaged, all samples from that line were dissociated with Accumax (Innovated Cell Tech.), counted on a Countess Automated Cell Counter (Invitrogen, C10227) using 0.4% trypan blue, and compared to the non-irradiated control group. Each bar on the graph (dose of radiation) is the average percent of control based on a minimum of three independent replicates (Error bars = ± SEM). The growth curve experiments following radiation presented in Additional file 3 were carried out in a similar manner with the following exceptions. All cell lines were plated at an initial density of 2 × 10^5^ cells per 3 ml and given a single 10 Gy dose of radiation or control (0 Gy). When the fastest growing sample was ready to be passaged, all cultures from each group were dissociated, counted, and then replated at 2 × 10^5^ cells. This was repeated three times in order to generate the growth curve presented in Additional file 3. Three IDH WT and three IDH1 mutant cultures were used for each group. Data is based on three independent replicates.

### IDH1 mutant overexpression/ROS measurement

cDNA for the IDH1 mutant gene (R132H) was cloned into a lentiviral vector and transfected into HK308 cell line. This vector encodes a murine orthologue and was found to maintain better and more consistent expression of the mutant protein and 2-HG than a human vector (data not shown). Five hundred thousand IDH1WT cells were dissociated and plated in 3 ml of neurosphere mediated, infected with the lentivirus, and allowed to grow for 2 weeks. The cells were sorted for GFP and again allowed to grow and form spheres. All experiments were done within 6 weeks of infection. Overexpression of the IDH1 mutant protein was confirmed by western blot and 2-HG measurement. IDH1 mutant and IDH wildtype cells were allowed to grow in gliomasphere media. They were then collected, stained with CellROX reagents (Thermo Fisher), and analyzed by flow cytometry. The total ROS level was the integration of the area under the curve as previously described [31].

### Statistical analysis

Statistical analyses were performed using the GraphPad Prism software. Sample comparisons and level of significance were determined using the ANOVA model and two-tailed Student’s *t* tests where appropriate. All quantitative data presented are the mean ± standard error of the mean (SEM) unless otherwise noted. Experiments were performed in triplicate, with calculation of 95% confidence interval and *p* values in relevant comparisons.

## Results

### KEGG GSEA analysis

**Fig. 1:**
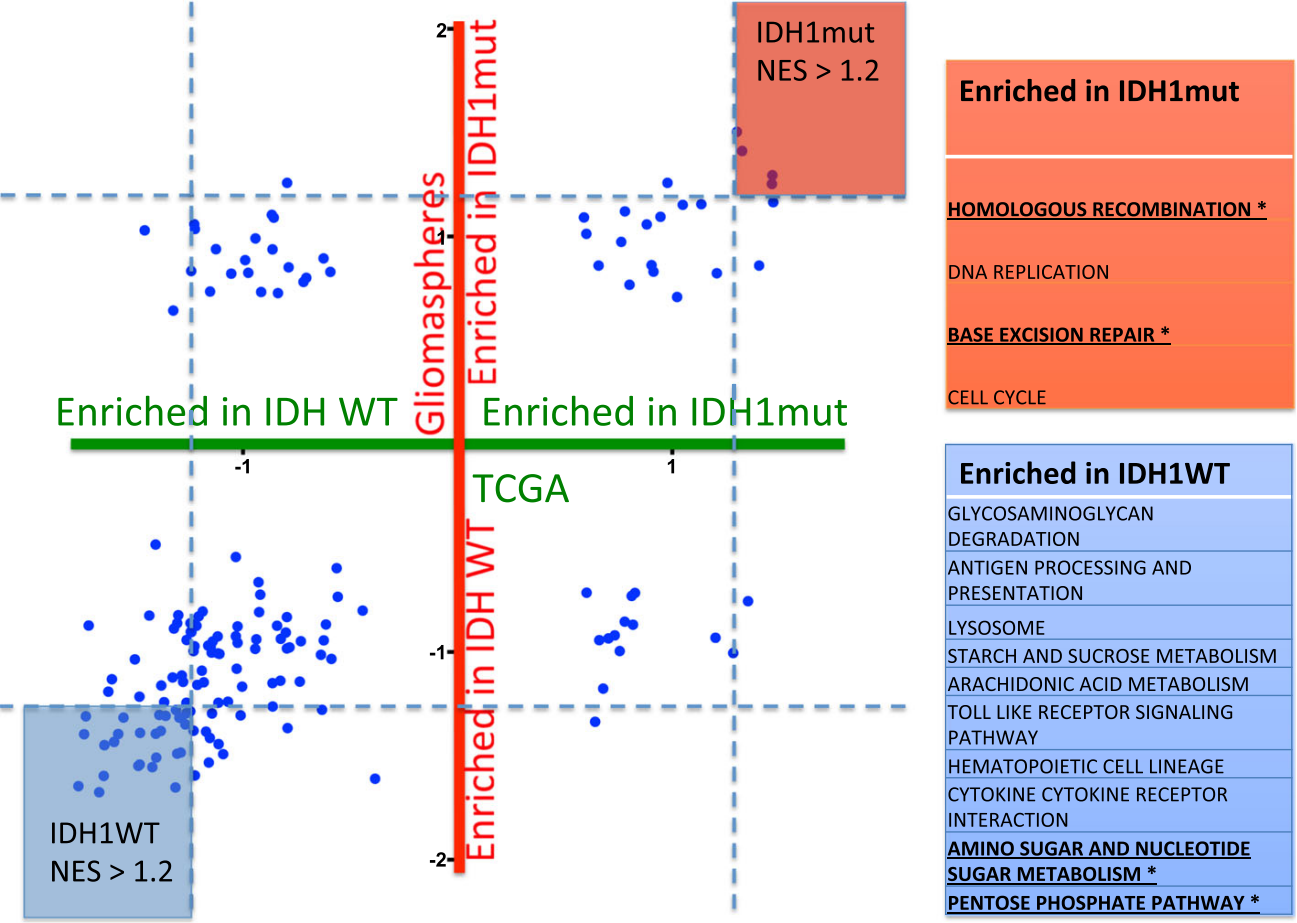
KEGG Gene Set Enrichment Analysis. Plot of expression data from TCGA (167 KEGG modules) and our gliomasphere dataset (186 KEGG modules). Each KEGG module was assigned a normalized enrichment score (NES) in either the IDH1 mutant or IDH1WT group. Each blue dot represents a different module. The enrichment score for a particular module in the TCGA data is plotted along the X-axis and that associated with the gliomasphere dataset for the same module is plotted on the Y-axis. Thus, a module that is highly enriched in IDH1 mutants compared to wildtype in both datasets would be in the upper right corner and modules highly enriched in IDH wildtype in both datasets would be in the lower left hand corner. Differentially enriched modules (NES > 1.2), listed on the right, were identified as potential candidates for further investigation

Expression data from 59 gliomasphere lines (52 IDH WT GBM and 7 IDH1 mutant) was subjected to Gene Set Enrichment Analysis (GSEA) using KEGG gene modules [32, 33]. Microarray data (GSE98995) is from data described in Laks et al. [17]. A similar comparative analysis was performed on IDH1 mutant and IDH WT tumor samples in the TCGA dataset (183 IDH1WT, 19 IDH1mut). Each KEGG module was assigned a normalized enrichment score (NES) for each dataset and then plotted (Fig. 1, Additional file 4). We noted a positive correlation between the gliomasphere and TCGA datasets giving confidence that our in vitro cells are a good model for in vivo tumors.

There were fewer modules enriched in the IDH1 mutant group in both the TCGA (37/167 gene set modules) as well as our gliomasphere data set (50/186 gene set modules). To identify some potential target metabolic pathways, we used a cut-off enrichment value of 1.2. Even with this liberal cut-off, we only identified four modules that were enriched in IDH1 mutant cells in both data sets. Of these four, the “Homologous Recombination” and “Nucleotide Base Excision Repair” modules were selected for further study due to the clinical relevance in terms of response to radiation. In contrast, there were 35 modules that were enriched in IDH WT cells (Fig. 1, Additional file 4). The “Pentose Phosphate Pathway” and “Amino Sugar and Nucleotide Sugar Metabolism” were selected for further study to determine if IDH1WT cells are in fact more dependent on the de novo pathway of nucleotide synthesis.

### Metabolic profile

To assess the differences found in the expression analysis, and to investigate any further metabolic differences between groups, a cohort of IDH WT and IDH1 mutant lines were selected for further study. Key mutations and CNVs of the five IDH1 mutant cultures and three IDH WT cultures that were most intensively studied are listed in Additional file 1. Of note, 4 of 5 of our IDH mutant cultures had pathogenic TP53 mutations and only one (BT142, obtained from ATCC) was 1p/19q co-deleted (Additional file 1). Sample 322 was lost prior to detailed analysis, but clinical cytogenetics of the primary tumor demonstrated that it was not 1p/19q co-deleted, despite it being an oligodendroglioma.

**Fig. 2:**
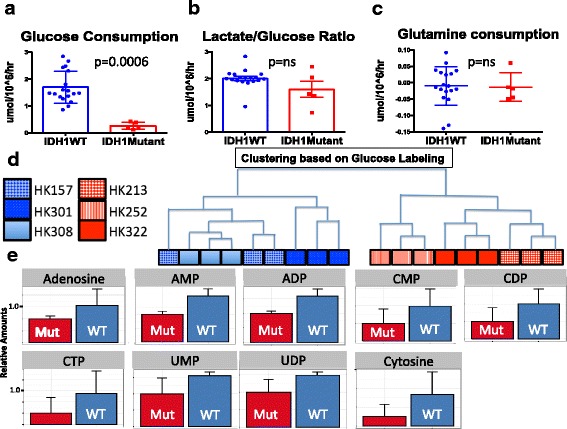
Metabolic profile of IDH1 mutant and wildtype cells. **a**–**c** Quantification of glucose and glutamine consumption as well as the ratio of glucose consumed to lactate produced. Measurements were acquired using NOVA (*N* = 18 IDH WT and 5 IDH1 mutant). Statistical significance was determined by Student’s *t* test. **d** Clustering of three IDH1 mutant and three IDH wildtype lines according to LC-MS glucose labeling (IDHWTs: blue, IDH1 mutants: red). A glucose labeling index for each of the 159 metabolites analyzed was calculated for all 6 cell lines followed by non-hierarchical clustering analysis. Each line was run in triplicate and all samples are shown. **e** Quantification of glucose labeling among nucleotide precursors by group. Relative amounts of metabolites were calculated by summing up all isotopologues of a given metabolite and normalized to the internal standard and cell number

A cohort of 18 IDH WT and 5 IDH1 mutant cultures were subjected to a panel of metabolic measurements including glucose uptake, glutamine uptake, and lactate production rates. Glucose uptake rate was significantly higher in IDH WT cells although there was no significant difference in the lactate production to glucose uptake ratio, suggesting that both cohorts are highly glycolytic. The net glutamine uptake rate for all cells tested was near zero (Fig. 2a–c). Consistent with these differences in glucose uptake, IDH wildtype cells grew faster than IDH1 mutant cells (Additional file 5).

To further define the different utilization of these metabolites, we performed LC-MS on three IDH WT (HK157, HK301, and HK308) and three IDH1 mutant (HK213, HK252, and HK322) lines with both fully labeled ^13^C glucose and fully labeled ^13^C glutamine. We then defined a single percent label for each metabolite and performed hierarchical clustering to identify if these six cell lines naturally partitioned into groups. Consistent with the observed differences in glucose uptake, IDH1 mutant and IDH WT samples partitioned into separate groups when assessed for glucose labeling (Fig. 2d). Applying hierarchical clustering to the samples according to glutamine labeling or total metabolite amount did not distinguish the samples into distinct groups.

Since glucose labeling could distinguish between IDH1 mutant and IDH WT samples, we performed *t* tests and compiled a list of all metabolites that had an uncorrected *p* value < 0.05. Thirty-two metabolites fulfilled this criterion of differential glucose labeling. Of these 32 metabolites, there was a significant over-representation of nucleotide precursors (9/32 chi-square *p* < 0.007). Interestingly, all of the nucleotide precursors showed increased glucose labeling in the IDH WT group (Fig. 2e, Additional file 6).

### De novo versus salvage nucleotide synthesis

**Fig. 3:**
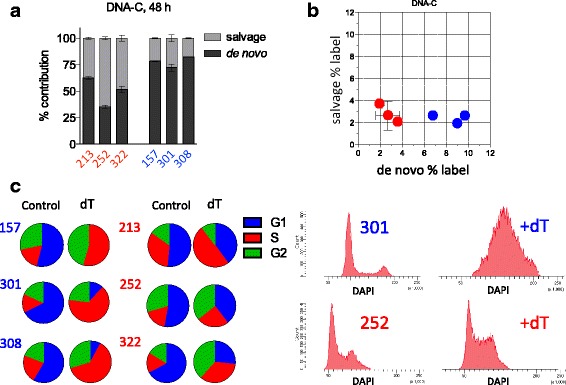
IDH1 mutant cells utilize de novo and salvage pathways for nucleotide biosynthesis. **a**, **b** Quantification of de novo nucleotide synthesis and salvage pathway utilization by LC-MS with labeled glucose and nucleotides. **a** The relative contribution of each pathway plotted in bar graph format. **b** The same data plotted two-dimensionally. Error bars represent ± STDEV. **c** Cell cycle analysis by flow cytometry (DAPI) following 4-day treatment with 1 mM deoxythymidine (dT) or control (left: distribution of cells in G1, S phase, and G2; right: representative histograms of cell cycle distribution for IDH1 mutant and WT cultures following dT treatment)

Because our gene expression and metabolomics results are consistent with elevated nucleotide metabolism in IDH WT cells, we decided to investigate further whether IDH WT cells are more dependent on de novo nucleotide synthesis than IDH1 mutant cells. We profiled the de novo and salvage contribution to deoxycytidine triphosphate incorporation into newly replicated DNA using labeled ^13^C glucose (to denote de novo) and labeled [^13^C9,^15^N3]dC (to denote salvage). Using the same set of three IDH WT lines and three IDH1 mutant lines, we found that while all samples utilized both pathways, the IDH WT samples used primarily de novo synthesis while the IDH1 mutant samples used both pathways relatively equally (Fig. 3a, b). To identify if this difference had functional relevance, we utilized high levels of deoxythymidine (dT), an inhibitor of the de novo pathway [34] to determine if there is any differential response between the two groups. Predicting that this inhibitor would have an effect on the ability of cells to pass through S phase, we treated cells for 4 days (~ 1 doubling time) with dT and then performed cell cycle analysis using DAPI. All cell lines showed an increase in the number of cells in S phase; however, in the IDH1 mutant samples, cells were better able to pass through S phase and proceed with cell division. In contrast, at the end of the 4-day treatment period, almost all IDH wildtype cells were found in the S phase (Fig. 3c).

### DNA repair in response to radiation

**Fig. 4:**
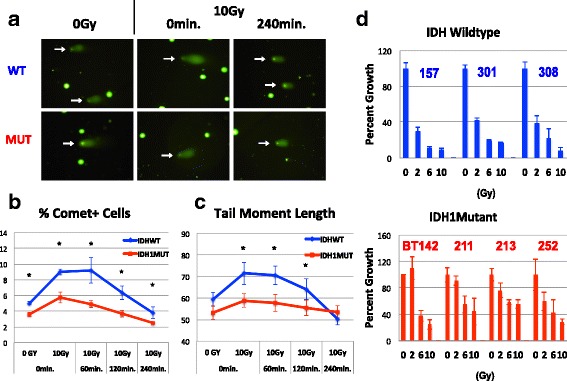
IDH1 mutant cells show diminished vulnerability to radiation. **a**–**c** Assessment of DNA damage and repair by comet analysis following irradiation (10 Gy). **a** Representative images of cells following comet assay. White arrows indicate cells with comets. **b** Graph showing the percent of cells with comets. Comet number was calculated for each line individually and then averaged by group. **c** Tail moment length for each comet was calculated as the length from the center of the comet head to the center of the tail. Tail moment length was calculated for each line individually and then averaged by group. Significance levels were calculated by ANOVA followed by post hoc t tests. Asterisks denote *p* < 0.05, error bars: ± SEM. **d** Quantification of the effects of increasing doses of radiation (0, 2, 6, and 10 Gy) on culture growth as described in the “Methods” section. Graphs represent cell counts normalized to non-irradiated controls

Having discovered that GSEA expression analysis accurately predicted that IDH1 mutant cells are less dependent on de novo nucleotide synthesis than are the IDH wildtype cells that we studied, we next turned to the modules that we found to be enriched in IDH1 mutant cells, namely modules involved with DNA repair and cellular response to radiation. Using a comet assay, we first tested whether there was a difference in the amount of DNA damage incurred from a given dose of radiation and how quickly that DNA was repaired. For these experiments, we studied two of our IDH1/TP53 mutant lines as well as BT142, a highly studied IDH1 mutant that is 1p/19q-co-deleted but that also bears a TP53 mutation [18, 19]. At a dose of 10 Gy, significantly more IDH WT cells showed signs of DNA damage as compared to IDH1 mutant cells at all time points tested (Fig. 4a, b). In IDH1 mutant cells, DNA damage peaked immediately after exposure to radiation, but that damage was largely repaired within 4 h. Damage in IDH wildtype cells peaked after the first hour post-radiation and followed a similar trend with regard to repair. To examine repair dynamics, we next restricted our focus only to those cells with sustained measurable damage (comet tails) and then calculated tail moment length at different time points (0, 60, 120, and 240 min) to assess which group more efficiently repairs DNA. Again, the IDH1 mutant cultures showed significantly less initial damage following radiation and were able to resolve DNA breaks more quickly (Fig. 4c). However, there was no significant difference in DNA damage between IDH1 mutant and wildtype cells by the final time point of the assay.

We assessed the extent of apoptotic cell death using the TUNEL assay but saw relatively low levels of TUNEL staining and no significant difference between the two groups (Additional file 7). However, when we examined the ability of cells to grow after radiation, we again found that IDH1 mutant cells were better able to re-enter the cell cycle and divide as compared to IDH WT cells (Fig. 4d). To ensure that these effects were not due to differences in growth rate between IDH1 mutant and wildtype cells, we repeated the experiment from Fig. 4d but performed a growth curve assay over several passages to compare effects of high dose radiation (10 Gy) on growth (Additional file 3). This analysis confirmed that IDH1 mutants once again grow better following radiation and are able to continue to proliferate after several passages. Meanwhile, IDH wildtype cultures are unable to sustain growth past the first week following this high dose of radiation. In all, these data suggest that the effects of radiation on growth are not simply due to differences in growth rates between IDH wildtypes and mutants.

### IDH1 mutant overexpression as a model for IDH1 mutant glioma

**Fig. 5:**
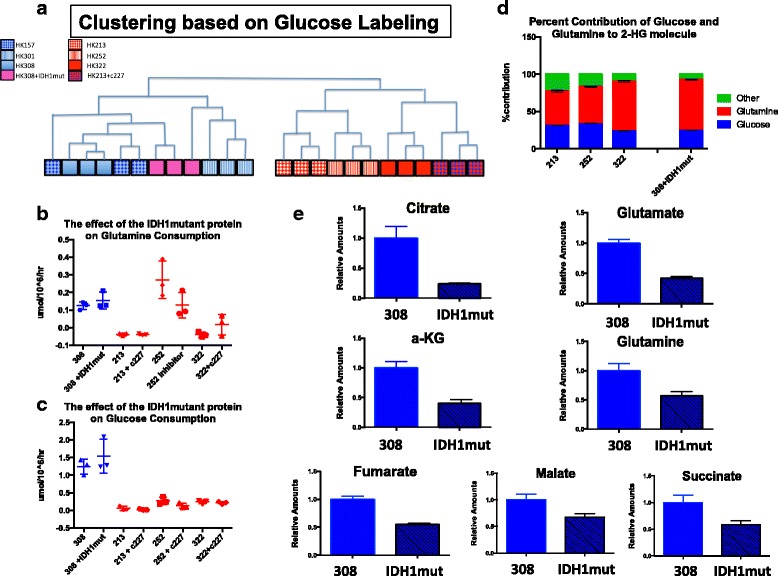
IDH1 mutant enzyme activity does not completely define the IDH1 mutant metabolic profile. **a** Clustering of eight samples based on LC-MS glucose labeling as described in Fig. 2. Analysis includes the same cohorts of IDH1 mutant and wildtype cultures with the addition of IDH1 mutant overexpression in an IDH WT line and inhibition of the endogenous mutant enzyme (308 + IDH1mut and 213 + c227 respectively). **b**, **c** Quantification of glutamine and glucose consumption with IDH1 mutant overexpression and inhibition. **d** Contribution of glucose and glutamine to 2-HG production in endogenous and transduced IDH1 mutants. **e** Effects of IDH1 mutant overexpression on glutamine and TCA cycle intermediates quantified using LC-MS. *p* < 0.05 for all seven metabolites shown. Error bars denote ± SEM

IDH1 mutant overexpression in an IDH1WT background makes those cells more vulnerable to radiation [7]. With our new result that endogenous IDH1 mutant cells are less vulnerable to radiation than the IDH WT cells tested, it appeared that the IDH1 mutation itself was not the sole determinant driving the difference between these two groups. To further test this hypothesis, we overexpressed the IDH1 mutant protein in an IDH WT background (HK308 + IDH1mut) and pharmacologically inhibited the IDH1 mutant protein in an endogenous IDH1 mutant cell line (HK213 + c227). We used LC-MS to confirm 2-HG production and inhibition respectively (Additional file 8). Once we confirmed the appropriate effects on 2-HG production, we repeated the glucose-uptake clustering analysis to see if this artificial IDH1 mutant line (HK308 + IDH1mut) clustered with the IDH WT or the IDH1 mutant groups (Fig. 5a). We found that the HK308 + IDH1mut samples clustered with the IDH WT group and the HK213 + c227 clustered with the IDH1mut group. More specifically, neither IDH1 mutant overexpression nor the c227 inhibitor led to a change in glucose labeling of nucleotide precursors (Additional file 9). This supports the hypothesis that the simple presence of IDH1 mutant protein or 2-HG was not driving the difference between these two groups over the time frames investigated.

We next investigated what effect the presence of the IDH1 mutant protein had on cellular metabolism. We examined the effects on glutamine and glucose consumption and found that neither the addition nor inhibition of the endogenous IDH1 mutant enzyme influenced the consumption of either metabolite (Fig. 5b, c). This was a surprising result given that the expression of the IDH1 mutant enzyme leads to production of high levels of 2-hydroxyglutarate and presumed consumption of alpha-ketoglutarate. To investigate this further, we used LC-MS tracing with labeled glucose and glutamine to determine how the cell makes 2-HG. Both endogenous IDH1 mutants and our overexpression model primarily used glutamine to make 2-HG (Fig. 5d). However, given that we did not observe an increase in the amount of glutamine consumption with the addition of the IDH1 mutant gene, we hypothesized that the cells may be depleted of glutamine. Consistent with this prediction, we saw significantly lower levels of glutamine as well as all TCA cycle intermediates when the IDH1 mutant gene was overexpressed (Fig. 5e). In a recent study, Li et al. found that elevating 2-HG levels leads to a significant accumulation of succinate with corresponding decreases in fumarate and malate [35]. Interestingly, we observed decreased levels of succinate and increases in fumarate and malate with 2-HG inhibition (c227) in an endogenous IDH1 mutant line (HK213). However, this did not accurately reflect the differences between endogenous IDH1 mutant and IDH wildtype cells which had roughly comparable levels of glutamine and TCA cycle intermediates (Additional file 10), suggesting that the IDH1 mutation is well-compensated for in the cells that carry it.

**Fig. 6:**
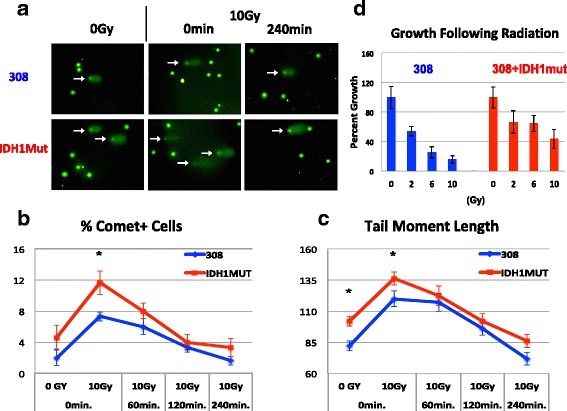
IDH1 mutant overexpression does not accurately mimic the DNA repair capacity of endogenous mutants. **a**–**c** Assessment of DNA damage and repair by comet analysis following radiation (10 Gy). **a** Representative images of cells following comet assay. White arrows point to cells with comets. **b** Graph shows the percent of cells with comets. Comet number was calculated for each line individually and then averaged by group. **c** Tail moment length for each comet is calculated as the length from the center of the comet head to the center of the tail. Tail moment length was calculated for each line individually and then averaged by group. **d** Quantification of the effects of increasing doses of radiation (0, 2, 6, and 10 Gy) on culture growth as described in the “Methods” section. Graphs represent cell counts normalized to non-irradiated controls. Significance levels were calculated by ANOVA followed by post hoc *t* tests. Asterisks denote *p* < 0.05, Error bars: ± SEM

In order to further identify potential differences between the endogenous IDH1 mutant and overexpression models, as well as to help clarify differences between our observations of radiation sensitivity from previous reports [7], we repeated our radiation experiments on HK308 with and without overexpression of the IDH1 mutant enzyme. Overexpression of the mutation in this IDH wildtype line resulted in greater DNA damage and decreased repair immediately following radiation (Fig. 6a–c). These results are contrary to what was observed when comparing endogenous IDH1 mutants to IDH wildtype cell lines (Fig. 4). IDH1 mutant overexpression did, however, result in better growth following radiation. One possible explanation for this finding is that exogenous expression of the IDH1 mutant enzyme in HK308 leads to significantly slower growth and this may give the cell more time to repair any DNA damage before re-entering the cell cycle. We also investigated the effects of IDH1 mutant inhibition (c227) on radiation-induced DNA damage and repair (Additional file 2). This analysis did not reveal any increase in DNA damage on a per cell basis, as measured by tail moment length. Interestingly though, we did see a more global increase in DNA damage in the c227-treated cells as shown by the increased number of comet+ cells. This treatment did not fully reverse the phenotype to that of IDH wildtype, however. One interpretation of these results is that endogenous IDH1 mutants are adapted to high levels of 2-HG which actually serve a protective role against DNA damage. On the other hand, IDH wildtype cells transduced with the mutant enzyme are not adapted to the dramatically increased levels of 2-HG, resulting in the opposite effect.

**Fig. 7:**
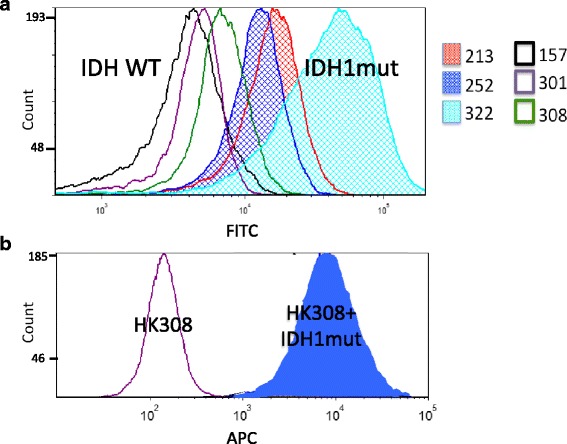
IDH1 mutant enzyme leads to higher ROS levels. **a** Histogram displaying ROS levels of three IDH1 mutant and three IDH1 wildtype gliomaspheres measured using flow cytometry (CellROX Green). **b** Similar ROS analysis of an IDH WT line (HK308) with and without IDH1 mutant overexpression (CellROX Deep Red). Results shown are from individual experiments. Three replicates yielded identical findings

Finally, we examined the effect of the IDH1 mutant enzyme on reactive oxygen species (ROS) levels. Interestingly, studies have reported mixed results regarding the effect of the IDH1 mutation on ROS with different results in different cell types [36, 37]. We found that the endogenous IDH1 mutant lines had significantly higher ROS levels than the IDH WT lines (Fig. 7a). This appears to be due to the IDH1 mutation itself, because when we overexpressed the IDH1 mutant enzyme in HK308, the ROS levels increased significantly (Fig. 7b).

## Discussion

Many groups have attempted to determine the effect of the IDH1 mutation on host cells as a way to discover metabolic vulnerabilities that may be exploited for therapy. Endogenous IDH1 mutant glioma cells are difficult to grow and maintain in culture—indeed, we lost one line during the process of these experiments—and thus, the first experiments to determine the effect of the IDH1 mutation on cells involved exogenous overexpression of the IDH1 mutant gene in an IDH WT background and performing mass spectroscopy to find differences in the levels of the various metabolites [13, 19, 38–40]. While many of these studies had overlapping findings (e.g., a decrease in glutamine and glutamate levels), other findings were more variable and seemed to depend on the cell model being used. Additionally, many of these in vitro differences did not translate to ex vivo patient tumor tissue [41].

Consistent with the aforementioned studies, we also saw a decrease in glutamine and glutamate with exogenous IDH1 mutant overexpression. However, there was no difference in glutamine, glutamate, or any TCA cycle intermediates between IDH wildtype cells and endogenous IDH1 mutant cells. This suggests that endogenous IDH1 mutant cells may be able to compensate for many of the effects of the IDH1 mutant enzyme itself. One possible explanation for this is that IDH1 mutant glioma cells and IDH wildtype glioma cells are derived from different cell types with different properties. However, it is also possible that given enough time, the exogenous IDH1 mutant model would also be able to compensate for the IDH1 mutant enzyme and increase its glutamine and glutamate levels.

Another approach to determine the metabolic effect of the IDH1 mutation on glioma cells is to inhibit the IDH1 mutation in an endogenous IDH1 mutant line. Both the current study and the prior study of Tateishi et al. [8] found a mixed effect of the IDH1 mutant inhibitor on TCA cycle intermediates, with citrate being significantly increased in both studies. In our study, we used a relatively short incubation period (24 h) with the c227 inhibitor in order to isolate the differences in metabolic flux from the changes in gene expression that are associated with 2-HG and aberrant methylation. Although the Tateishi study did not find any significant changes in global methylation with prolonged c227 inhibitor treatment, they did find an increase in NADH levels likely due to increased Naprt1 expression and NAD synthesis. We did not see this change over our shorter time course, and in fact, we saw decreased NADH levels with c227 treatment.

A current challenge in IDH mutant research stems from the variability in methodological approaches used which can significantly alter findings. Identifying potential shortcomings of each will greatly improve the reliability of results. For example, by restricting comparisons to purely isogenic cell lines with or without IDH1 mutant enzyme overexpression or inhibition, the conclusions are limited to the effect of the IDH1 mutant enzyme itself and ignore differences between IDH1 mutant and IDH wildtype tumors that may be independent of the IDH1 mutation. Furthermore, metabolites do not act independently but function as parts of larger pathways. Using only the total cellular levels of various metabolites, it is difficult to draw any conclusions about particular pathways. In this study, we utilized multiple methods to identify cell-type specific vulnerabilities. By using expression data and metabolic tracing studies, we were readily able to distinguish several pathways as well as labeling patterns that clearly distinguished IDH1 mutant from IDH wildtype cells. This distinctive metabolic signature was intrinsic to the cell itself, was retained even with pharmacological inhibition of the IDH1 mutant enzyme, and was not seen in the exogenous overexpression model.

Further evidence of cell culture model variability between IDH mutant studies can be seen from two additional reports published during the preparation of this manuscript, Sulkowski et al. and Lu et al. [42, 43], which demonstrated a diminished capacity for DNA damage repair in IDH1 mutant cells citing deficiencies in homologous recombination and compromised PARP-mediated DNA repair. We observed greater DNA damage and a similar decreased capacity for DNA repair only when using an IDH1 mutant overexpression model. In contrast, our results indicate greater DNA repair capacity in IDH1 mutant cells following radiation. One potential explanation could be that our cultures represent only a subset of IDH1 mutant tumors. Most, if not all, contain endogenous IDH1 R132H mutations and mutant TP53, an important protein that itself may play a large role in mediating many of the observed effects independent of the IDH1 mutant enzyme. Furthermore, the IDH wildtype GBM cultures that were selected as controls could influence the differences observed. While both of these prior studies utilize exogenously transduced IDH mutations, which may have different characteristics, one report did also utilize glioma cells with endogenous mutations for some of their key experiments [42]. However, it is not clear whether these mutant lines are similar to ours with respect to their other mutations. Furthermore, the endogenous mutant cultures from the prior report were propagated in serum, while ours are continuously maintained in serum-free conditions. The differences among these studies have important ramifications for therapy. If, as we propose, IDH1 mutants have a greater capacity for DNA repair following radiation, then radiation therapy may have diminished efficacy. Further study will be required to resolve these differences.

Many of the clinical trials that guide current treatment protocols for glioblastoma were conducted before the discovery of the IDH mutation and thus included a majority of IDH wildtype and a minority of IDH mutant patients. With evidence mounting that IDH mutant gliomas have very different cellular characteristics, it is unclear whether the results of these trials can be generalized to IDH mutant tumors. In particular, the results of this study show that IDH1 mutant gliomas are less vulnerable to radiation-induced DNA damage which draws into question the efficacy of radiation therapy for this subset of tumors. This result was surprising when combined with the fact that exogenous IDH1 mutant overexpression makes cells more vulnerable to radiation [7] and that the IDH1 mutant enzyme significantly increases the amount of reactive oxygen species in the cell. While the cytotoxic and cytostatic mechanisms of radiation are multi-factorial, one of the main mechanisms is DNA damage through the indirect production of free radicals [44]. One possible explanation for the decreased DNA damage seen in IDH mutant cells following radiation is that in order to survive this mutation, IDH mutant cells must develop buffering mechanisms against high levels of reactive oxygen species. This adaptation in turn could make them more resistant to radiation induced DNA damage.

The clinical evidence for the efficacy of radiation in IDH1 mutant gliomas is mixed. In one study, IDH1 mutant tumors seemed to show a better radiographic response following radiation [45]. However, another trial found that for supratentorial low-grade gliomas (of which the majority are likely IDH mutant) high-dose radiation had worse survival than low-dose radiation [46]. It is possible that both findings are correct and that radiographic response may not correlate with clinical survival. Conversely, it is possible that IDH mutant tumors are maximally sensitive to low-dose radiation and higher doses do not provide any further benefit.

Our studies of nucleotide metabolism have potential implications for therapeutics. The availability of nucleotides for DNA synthesis can be a rate-limiting step in cellular proliferation [47]. Nucleotides are synthesized through either de novo or *salvage pathways*. Pharmacologically, the de novo pathway can be inhibited by high concentrations of dT and the salvage pathway can be inhibited by a novel class of compounds [34]. Our data suggest that IDH1 mutant tumors, in contrast to the majority of GBM, utilize this salvage pathway and would hence require both pathways to be inhibited in therapeutic strategies employing inhibition of nucleotide biosynthesis.

A limitation to the current study is that, while gene expression studies are performed using both cultured cells and data derived directly from IDH mutant tumors in vivo, our functional results are obtained from in vitro studies. Very few studies have investigated a spectrum of IDH mutant gliomas in vivo. This is undoubtedly due to the difficulty in propagating these cells in xenograft models. Although not shown, the cells we have utilized did not form xenografts in immunodeficient mice, either in the brain or subcutaneously. The reasons for this are unknown, but suggest that the establishment of tumors from endogenous IDH1 mutants requires host-derived factors that are not present in our model systems. While our findings present novel hypotheses and avenues for future studies, some caution must be taken prior to applying our results directly to clinical trials.

## Conclusions

With evidence mounting that IDH mutant gliomas constitute a distinct subclass that follows an independent path of tumorigenesis [48], we endeavored to characterize metabolic differences between IDH1 mutant and IDH wildtype gliomas. Our data are consistent with this concept and provide evidence that some of the distinctions may not be directly related to the IDH1 mutation itself. Furthermore, our data suggest that while IDH1 mutant gliomas may have a less malignant phenotype, they may also be relatively resistant to certain therapies, including radiation.

## Additional files

Additional file 1: Patient-derived gliomasphere culture characteristics. Relevant patient information and clinical characteristics for the primary, patient-derived gliomasphere cultures used in this study. Summary of selected copy-number alterations and mutations are also shown. (PDF 42 kb)

Additional file 2: 2-HG inhibition with c227 moderately increases DNA damage following radiation but does not reverse the IDH1 mutant phenotype to an IDH wildtype phenotype. A-B. Assessment of DNA damage and repair by comet analysis following irradiation (10 Gy). HK252 was treated for 48 h with 5 μM c227 or control prior to comet analysis and compared to the IDH WT line HK157. A. Similar analysis of tail moment length as conducted in Fig. 4. B. Graph showing the percent of cells with comets as explained in Fig. 4. All error bars represent ± SEM. (PDF 46 kb)

Additional file 3: IDH1 mutant cells are better able to proliferate following radiation than IDH wildtype cells. A. Growth curve following radiation (0 and 10 Gy) shows average fold increases in cell number between IDH wildtype and IDH1 mutant groups over three passages. Each group consists of three IDH1 mutant and three IDH wildtype cultures respectively. Growth curves were generated from individual cell counts at each time point. B. Identical growth curve as shown in (A), however, the non-irradiated groups have been removed for better visual comparison between irradiated IDH mutant and wildtype groups. Error bars represent ± STDEV. (PDF 51 kb)

Additional file 4: KEGG gene set enrichment analysis of IDH1 mutant and wildtype gliomaspheres. Thirty-five modules were enriched in IDH wildtypes compared to four modules in IDH1 mutants. (PDF 79 kb)

Additional file 5: IDH wildtype cells show faster growth rate compared to IDH1 mutant cells. Six IDH wildtype and five IDH1 mutant gliomasphere cultures were assessed for proliferation rate by flow cytometry using carboxyfluorescein succinimidyl ester (CFSE). (*p* < 0.05). (PDF 19 kb)

Additional file 6: Glucose labeling of metabolites. Glucose fractional contribution was computed for all 159 metabolites. Metabolites that were significantly different between groups (uncorrected *t* test *p* < 0.05) are shown here. (PDF 137 kb)

Additional file 7: Radiation leads to low levels of TUNEL staining in gliomaspheres. Representative images and quantification of cells stained for TUNEL positivity 4 days after exposure to increasing doses of radiation. There is no significant difference between groups. Error bars represent ± SEM. (PDF 2212 kb)

Additional file 8: IDH1 mutant overexpression leads to high levels of 2-HG and the c227 inhibitor is an effective inhibitor of 2-HG formation. IDH wildtype gliomaspheres transduced with the IDH1 mutant enzyme (308 + IDH1mut) and endogenous IDH1 mutant cells treated with 5 μM c227 inhibitor (213 + c227) for 24 h are compared to their respective controls (308 and 213) for 2-HG levels as determined by LC-MS. Data represent the means ± SEM of three replicates per condition. (PDF 22 kb)

Additional file 9: IDH1 mutant enzyme does not affect glucose labeling of nucleotide precursors. Cells were treated and analyzed as described for Supporting Figure S4 in order to determine the percent labeling of nucleotide precursors. There is no significant difference when comparing IDH1 mutant overexpression or endogenous mutant inhibition to their respective controls. (PDF 64 kb)

Additional file 10: Effects of pharmacologic inhibition of the IDH1 mutant enzyme on TCA cycle intermediates. Cells were analyzed as described in Supporting Figure S5. Left: Metabolites with significantly different percent glucose labeling of metabolites in the endogenous IDH1 mutant line 213 treated with c227 inhibitor or control (*p* < 0.05). Right: Percent labeling from endogenous IDH1 mutant and IDH wildtype groups for metabolites that were not significantly different. (PDF 206 kb)

## Abbreviations

2-HG: 2-hydroxyglutarate
a-KG: Alpha-ketoglutarate
dT: Deoxythymidine
GSEA: Gene set enrichment analysis
IDH: Isocitrate dehydrogenase
LC-MS: Liquid chromatography–mass spectrometry
PARP: Poly ADP ribose polymerase
ROS: Reactive oxygen species
TCA cycle: Tricarboxylic acid cycle
TP53: Tumor protein 53
