# Supplementary material for: Metabolic characterization of isocitrate dehydrogenase (IDH) mutant and IDH wildtype gliomaspheres uncovers cell type-specific vulnerabilities
Source: Cancer Metab. 2018 Apr 17;6:4. doi: 10.1186/s40170-018-0177-4 (PMC5905129; doi:10.1186/s40170-018-0177-4)
Supplement: Supplementary file 1 — Patient-derived gliomasphere culture characteristics. Relevant patient information and clinical characteristics for the primary, patient-derived gliomasphere cultures used in this study. Summary of selected copy-number alterations and mutations are also shown. (PDF 42 kb) [file 40170_2018_177_MOESM1_ESM.pdf]

Culture ID	Patient Age	Patient Sex	Diagnosis	Treatment	IDH1 Status	1p/19q co-deleted	EGFR	TP53	PTEN	ATRX	CDKN2A
HK211	41	F	Secondary GBM	N/A	p.R132H/ p.V178I	No	**vIII**	p.N235S/ p.Y234C			
HK213*	39	M	Secondary GBM	XRT, TMZ	p.R132H	No		p.P250L			
HK252*	40	M	Secondary GBM	XRT, TMZ	p.R132H	No		p.P250L			
HK322	37	F	Oligodendrogli- oma Grade II	N/A	p.R132H	No	CN Unknown	CN Unknown	CN Unknown	CN Unknown	CN Unknown
BT142**	38	M	Oligoastrocytom a Grade III	N/A	p.R132H	Yes		p.I50fs			
HK157	54	F	Primary GBM	N/A	WT	No		WT			
HK301	65	M	Primary GBM	N/A	WT	No	**vIII**	WT			
HK308	50	F	Recurrent GBM	XRT, TMZ, Avastin	WT	No	CN Unknown (**vIII**)	CN Unknown (p.A161T)	CN Unknown	CN Unknown	CN Unknown

*Different resections from same patient

**Obtained from ATCC

vIII- EGFR variant III mutant

Copy Number (CN)

CN Gain

CN Loss (-1)

CN Loss (-2)
