# Supplementary figures and images for: Metabolic characterization of isocitrate dehydrogenase (IDH) mutant and IDH wildtype gliomaspheres uncovers cell type-specific vulnerabilities
Source: Cancer Metab. 2018 Apr 17;6:4. doi: 10.1186/s40170-018-0177-4 (PMC5905129; doi:10.1186/s40170-018-0177-4)

**A.****Tail Moment Length**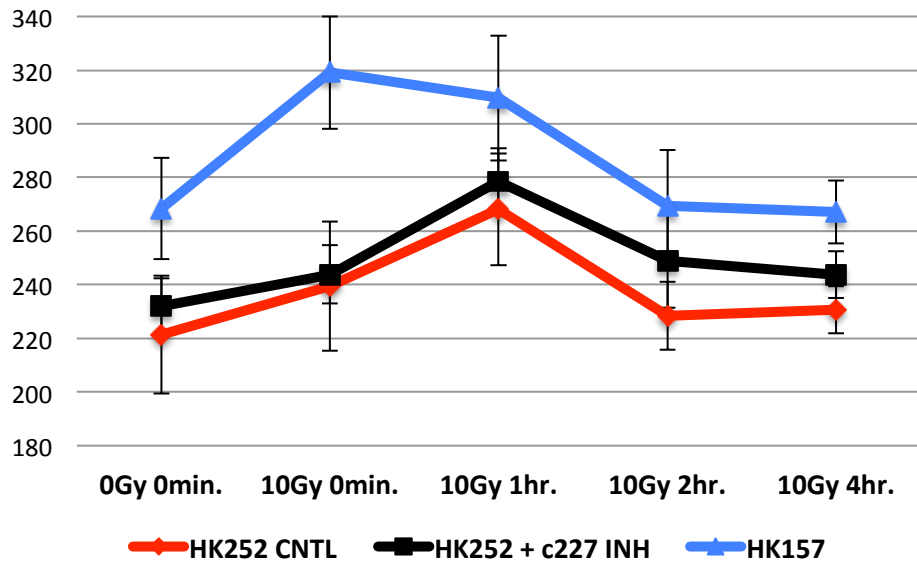**B.****% Comet+ Cells**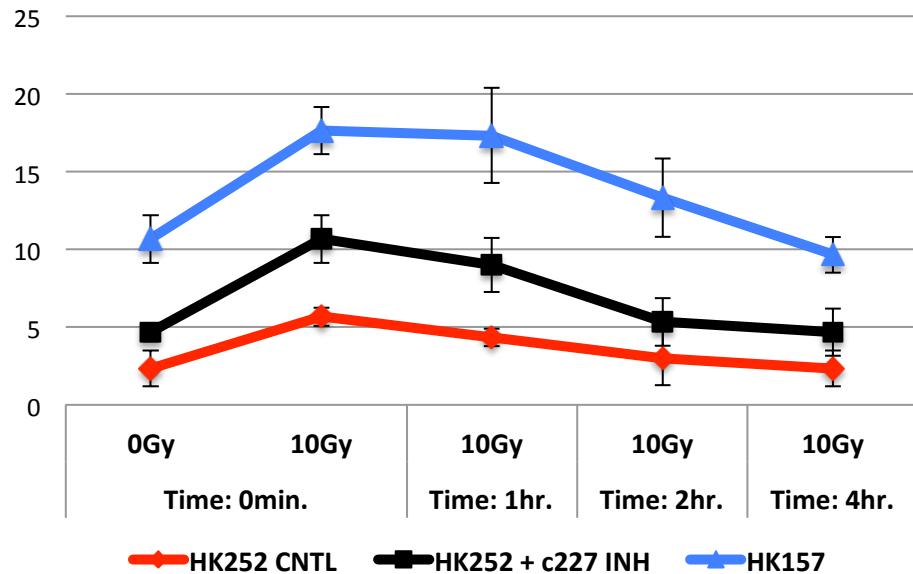

Supplement: Supplementary file 2 — 2-HG inhibition with c227 moderately increases DNA damage following radiation but does not reverse the IDH1 mutant phenotype to an IDH wildtype phenotype. A-B. Assessment of DNA damage and repair by comet analysis following irradiation (10 Gy). HK252 was treated for 48 h with 5 μM c227 or control prior to comet analysis and compared to the IDH WT line HK157. A. Similar analysis of tail moment length as conducted in Fig. 4. B. Graph showing the percent of cells with comets as explained in Fig. 4. All error bars represent ± SEM. (PDF 46 kb) [file 40170_2018_177_MOESM2_ESM.pdf]

**A.****Growth Following 10Gy Radiation**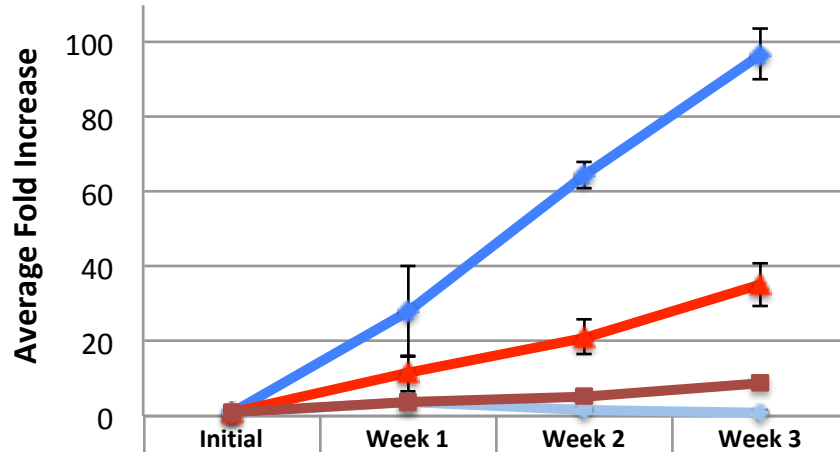**B.****Growth Following 10Gy Radiation**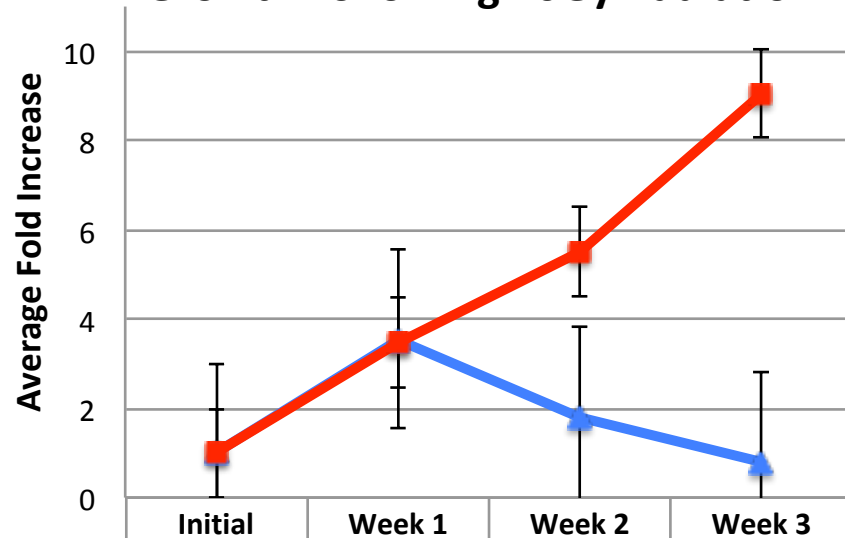

|          |   |             |             |         |
|----------|---|-------------|-------------|---------|
| WT 10GY  | 1 | 3.555925926 | 1.825704561 | 0.8     |
| MUT 10GY | 1 | 3.464814815 | 5.508888889 | 9.05654 |

Supplement: Supplementary file 3 — IDH1 mutant cells are better able to proliferate following radiation than IDH wildtype cells. A. Growth curve following radiation (0 and 10 Gy) shows average fold increases in cell number between IDH wildtype and IDH1 mutant groups over three passages. Each group consists of three IDH1 mutant and three IDH wildtype cultures respectively. Growth curves were generated from individual cell counts at each time point. B. Identical growth curve as shown in (A), however, the non-irradiated groups have been removed for better visual comparison between irradiated IDH mutant and wildtype groups. Error bars represent ± STDEV. (PDF 51 kb) [file 40170_2018_177_MOESM3_ESM.pdf]

# Division time IDH1WT vrs. IDH1mut

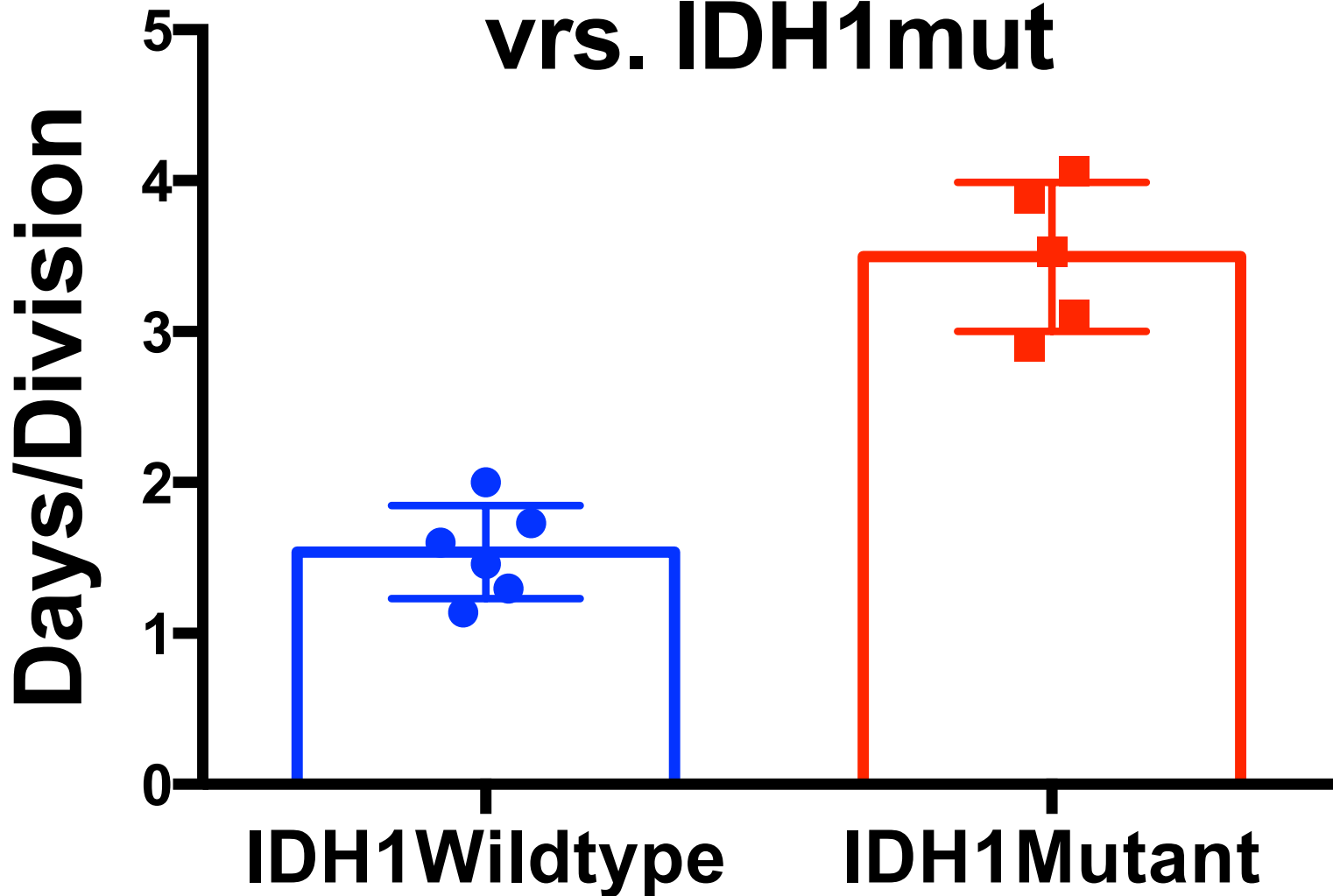

Supplement: Supplementary file 5 — IDH wildtype cells show faster growth rate compared to IDH1 mutant cells. Six IDH wildtype and five IDH1 mutant gliomasphere cultures were assessed for proliferation rate by flow cytometry using carboxyfluorescein succinimidyl ester (CFSE). (p < 0.05). (PDF 19 kb) [file 40170_2018_177_MOESM5_ESM.pdf]

# Glucose Labeling of Metabolites (p<0.05)

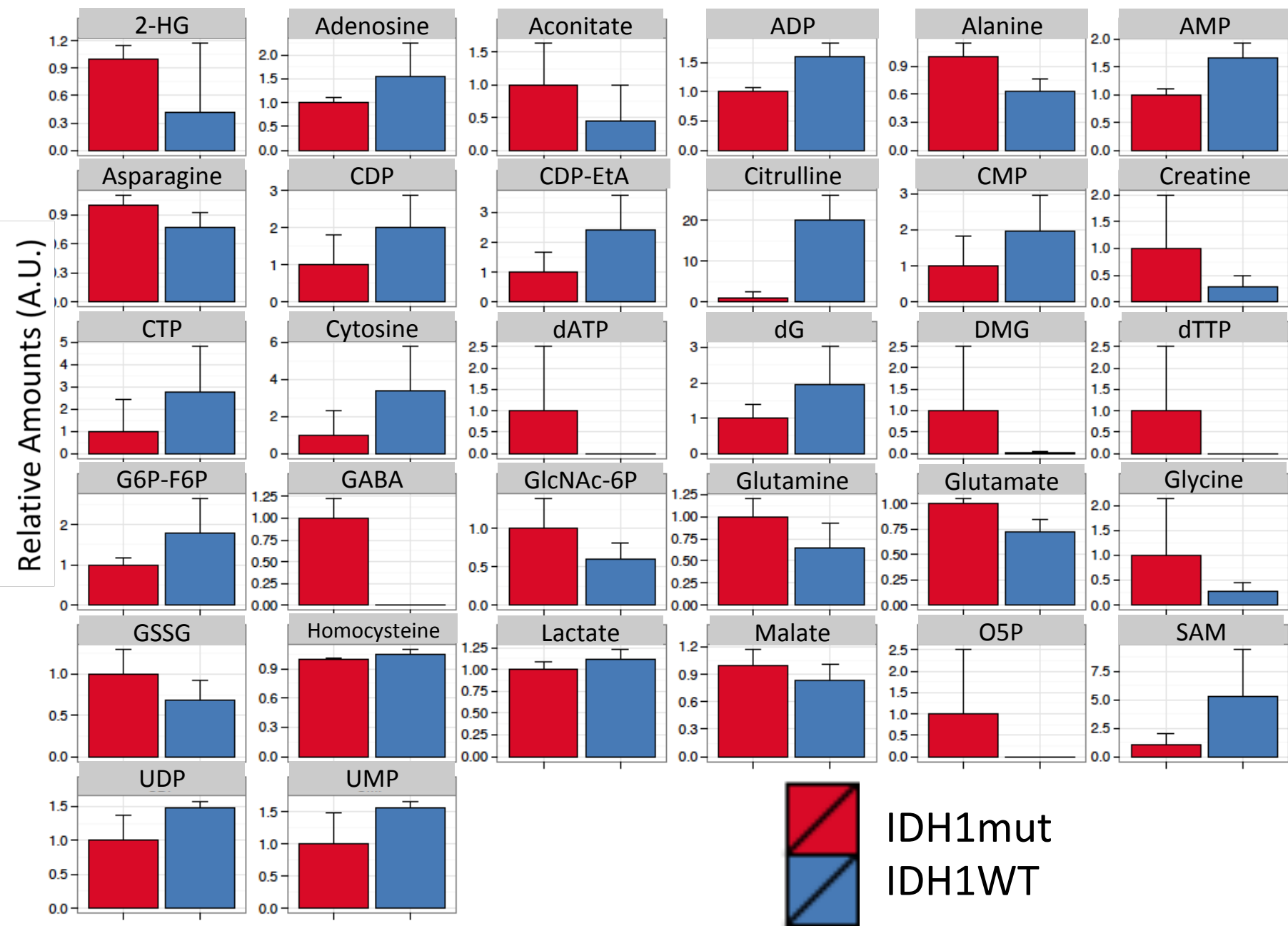

Supplement: Supplementary file 6 — Glucose labeling of metabolites. Glucose fractional contribution was computed for all 159 metabolites. Metabolites that were significantly different between groups (uncorrected t test p < 0.05) are shown here. (PDF 137 kb) [file 40170_2018_177_MOESM6_ESM.pdf]

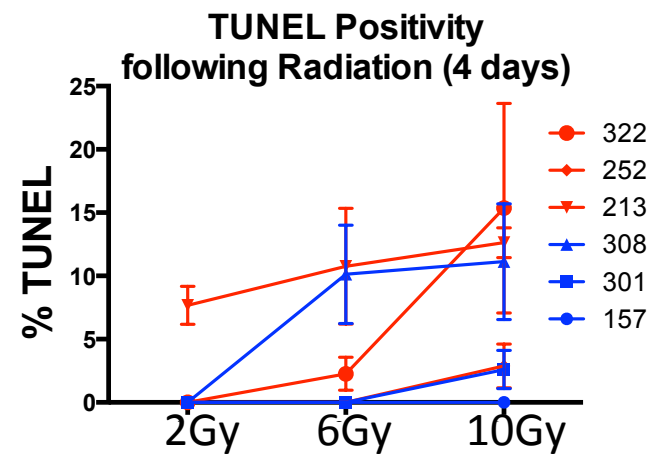

157

301

308

213

252

322

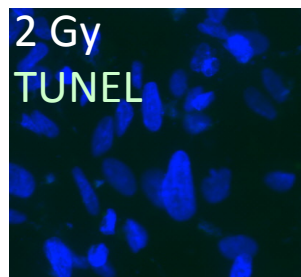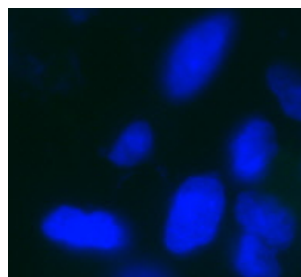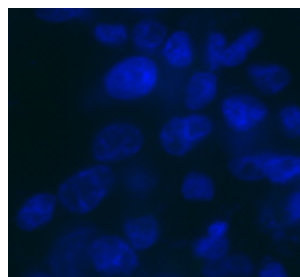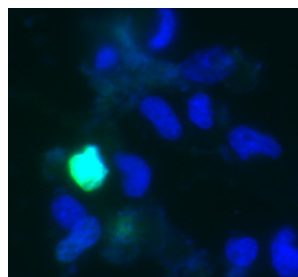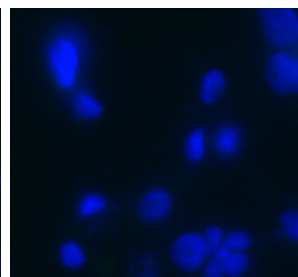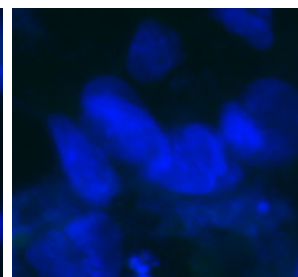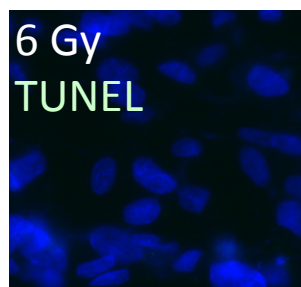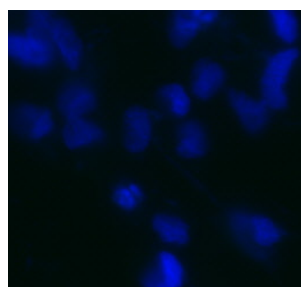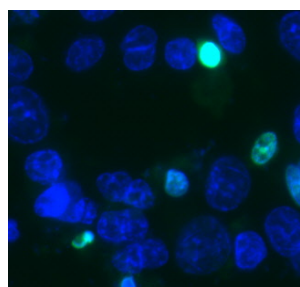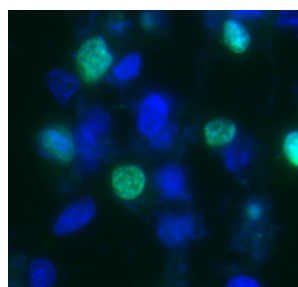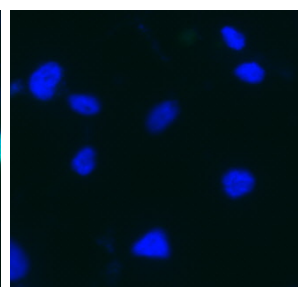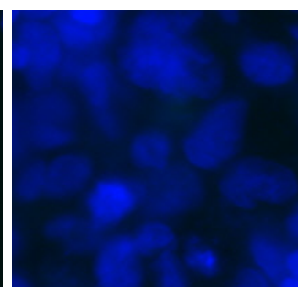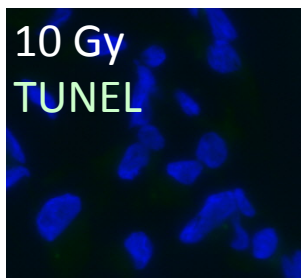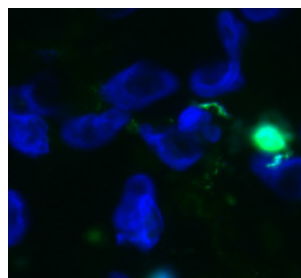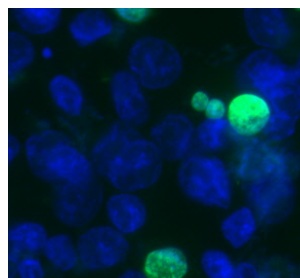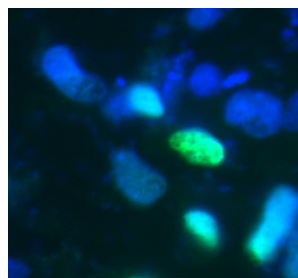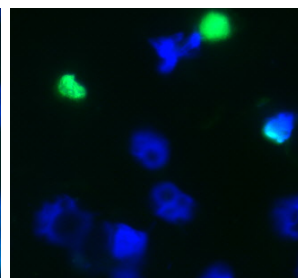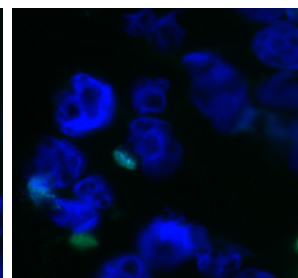

Supplement: Supplementary file 7 — Radiation leads to low levels of TUNEL staining in gliomaspheres. Representative images and quantification of cells stained for TUNEL positivity 4 days after exposure to increasing doses of radiation. There is no significant difference between groups. Error bars represent ± SEM. (PDF 2212 kb) [file 40170_2018_177_MOESM7_ESM.pdf]

# 2-hydroxyglutarate levels

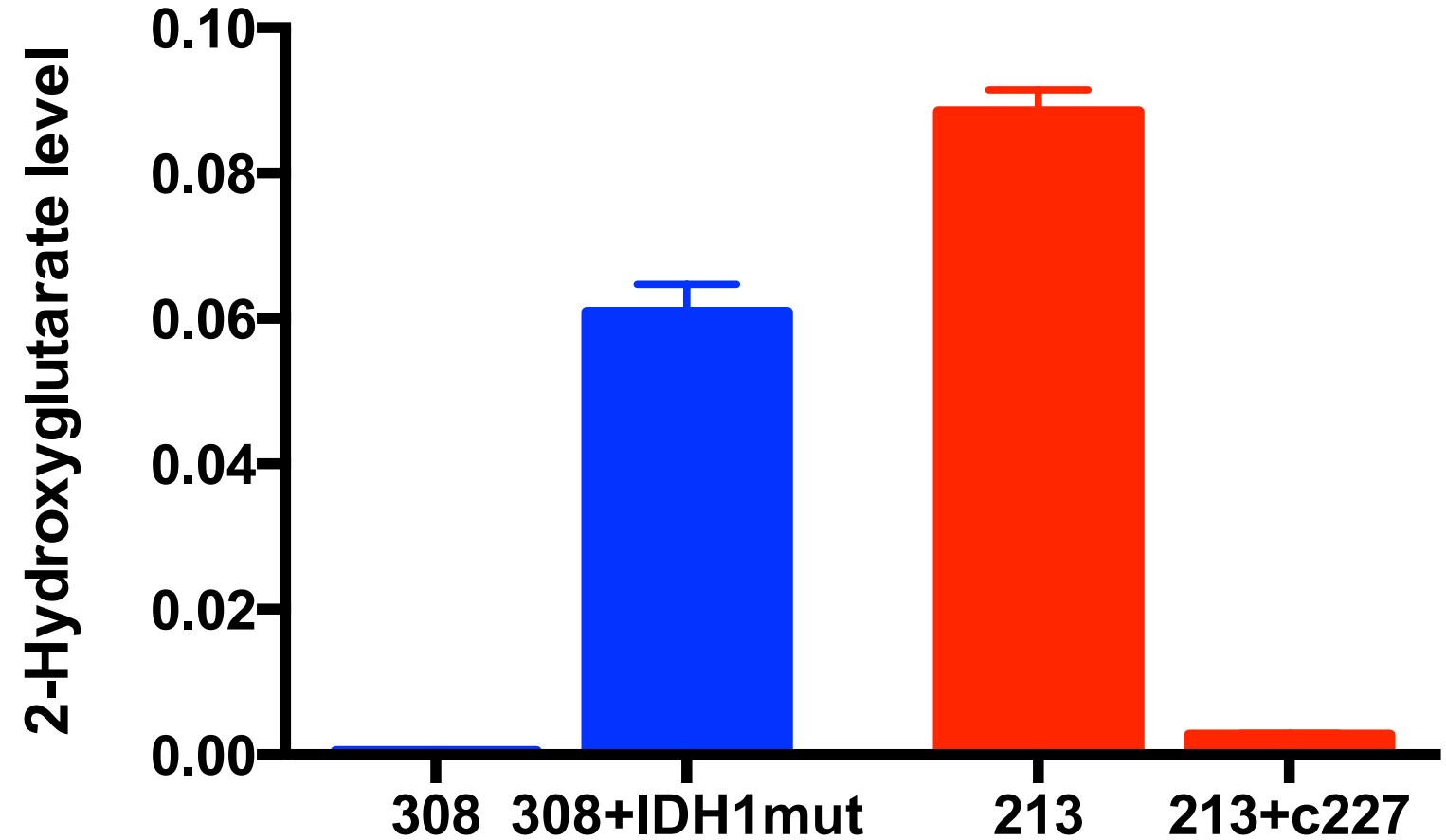

Supplement: Supplementary file 8 — IDH1 mutant overexpression leads to high levels of 2-HG and the c227 inhibitor is an effective inhibitor of 2-HG formation. IDH wildtype gliomaspheres transduced with the IDH1 mutant enzyme (308 + IDH1mut) and endogenous IDH1 mutant cells treated with 5 μM c227 inhibitor (213 + c227) for 24 h are compared to their respective controls (308 and 213) for 2-HG levels as determined by LC-MS. Data represent the means ± SEM of three replicates per condition. (PDF 22 kb) [file 40170_2018_177_MOESM8_ESM.pdf]

# %Glucose Label of Nucleotide Precursors

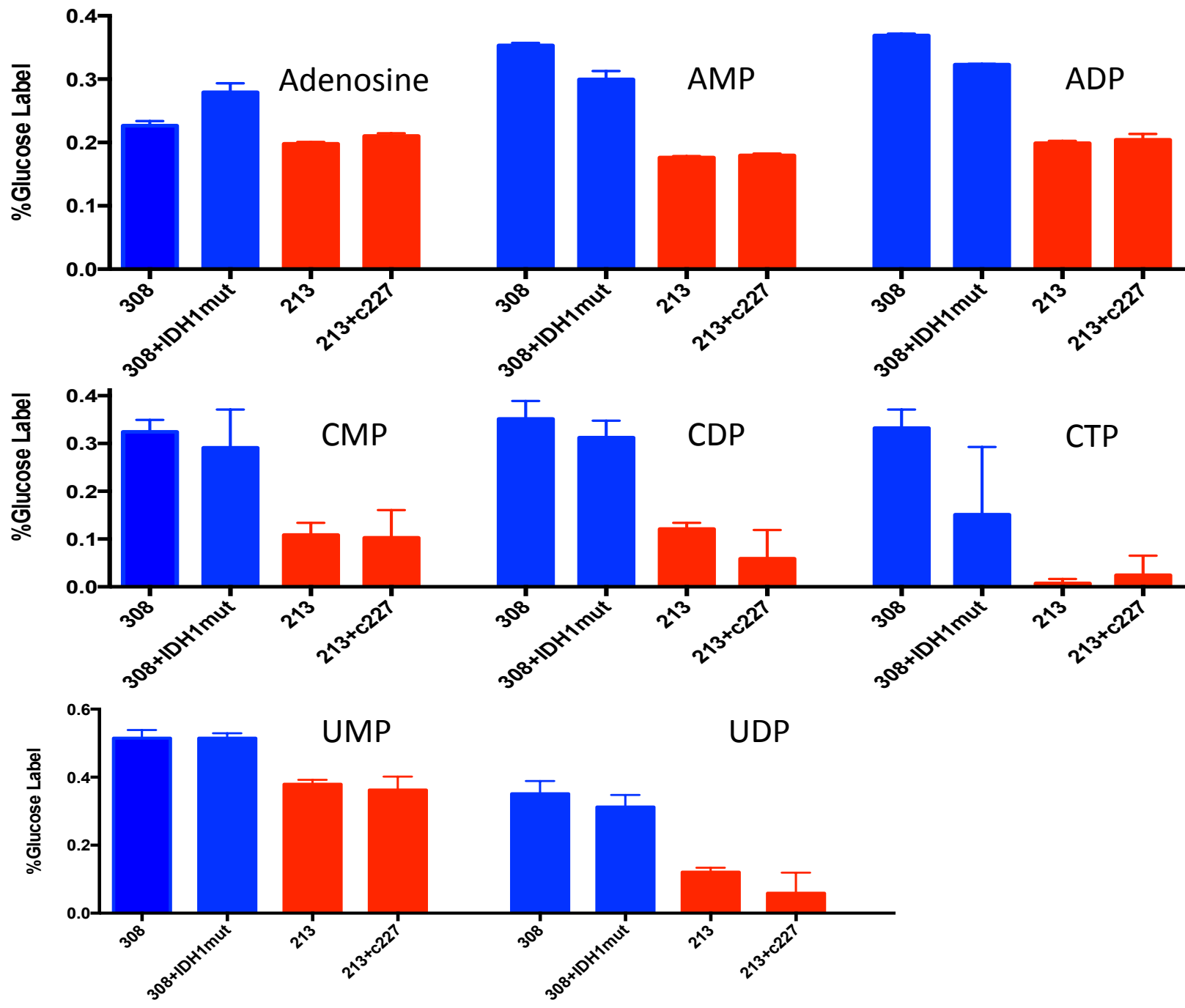

Supplement: Supplementary file 9 — IDH1 mutant enzyme does not affect glucose labeling of nucleotide precursors. Cells were treated and analyzed as described for Supporting Figure S4 in order to determine the percent labeling of nucleotide precursors. There is no significant difference when comparing IDH1 mutant overexpression or endogenous mutant inhibition to their respective controls. (PDF 64 kb) [file 40170_2018_177_MOESM9_ESM.pdf]

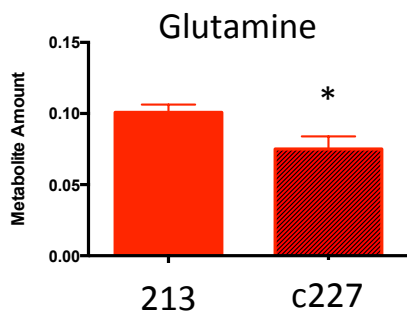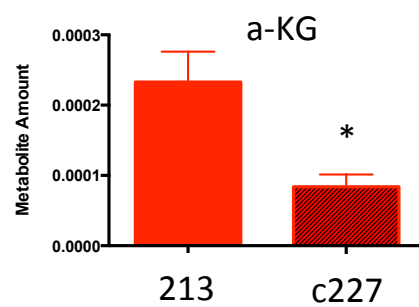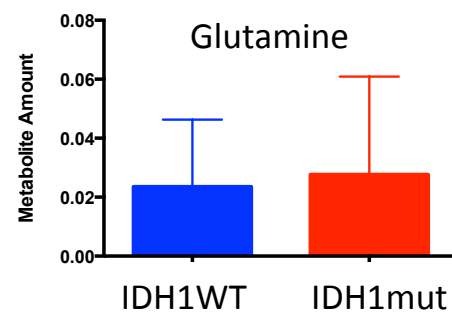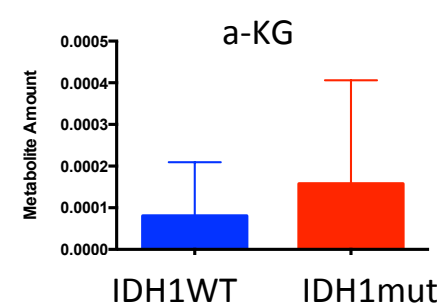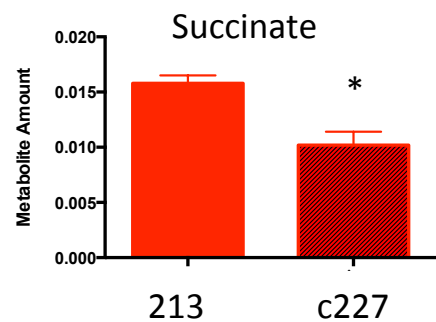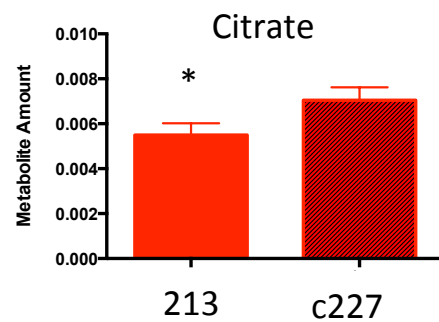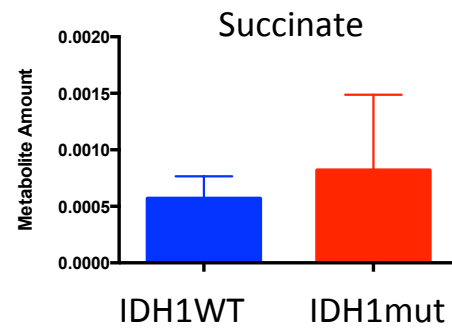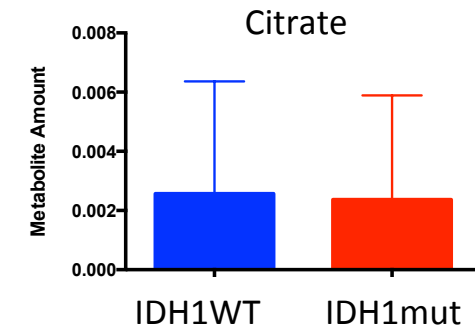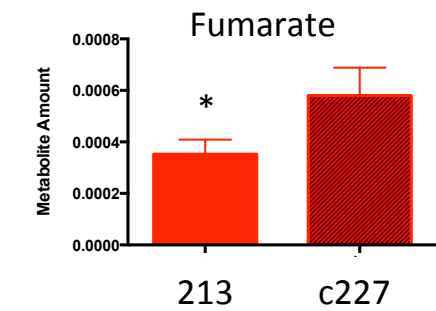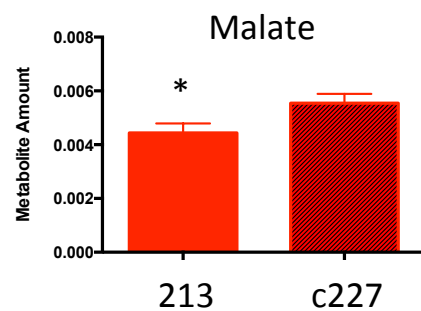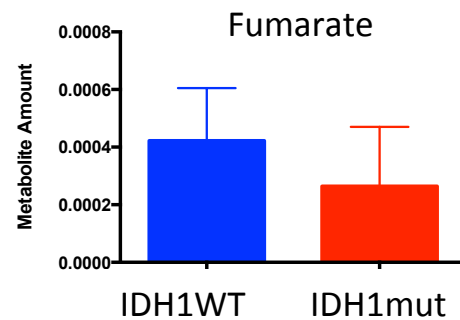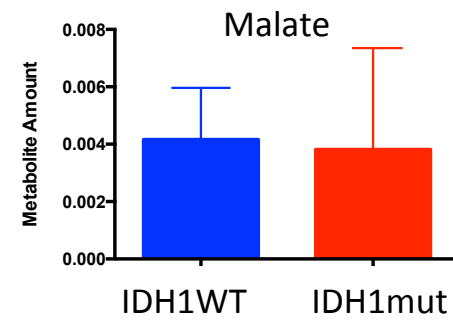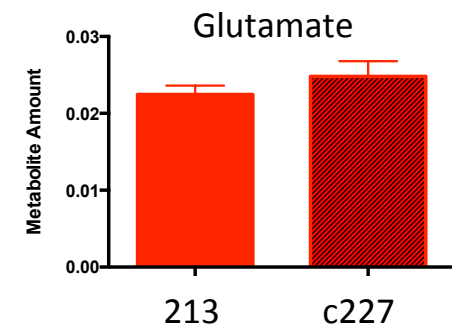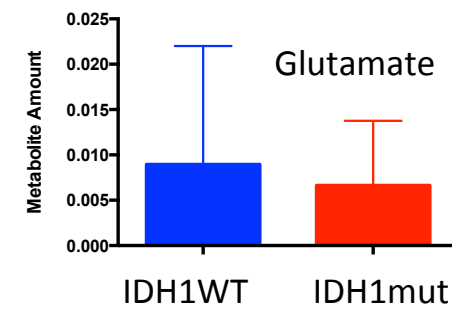

Supplement: Supplementary file 10 — Effects of pharmacologic inhibition of the IDH1 mutant enzyme on TCA cycle intermediates. Cells were analyzed as described in Supporting Figure S5. Left: Metabolites with significantly different percent glucose labeling of metabolites in the endogenous IDH1 mutant line 213 treated with c227 inhibitor or control (p < 0.05). Right: Percent labeling from endogenous IDH1 mutant and IDH wildtype groups for metabolites that were not significantly different. (PDF 206 kb) [file 40170_2018_177_MOESM10_ESM.pdf]
