# Supplementary material for: Metabolic characterization of isocitrate dehydrogenase (IDH) mutant and IDH wildtype gliomaspheres uncovers cell type-specific vulnerabilities
Source: Cancer Metab. 2018 Apr 17;6:4. doi: 10.1186/s40170-018-0177-4 (PMC5905129; doi:10.1186/s40170-018-0177-4)
Supplement: Supplementary file 4 — KEGG gene set enrichment analysis of IDH1 mutant and wildtype gliomaspheres. Thirty-five modules were enriched in IDH wildtypes compared to four modules in IDH1 mutants. (PDF 79 kb) [file 40170_2018_177_MOESM4_ESM.pdf]

Enriched in IDH1WT	
GLYCOSAMINOGLYCAN DEGRADATION	APOPTOSIS
ANTIGEN PROCESSING AND PRESENTATION	NATURAL KILLER CELL MEDIATED CYTOTOXICITY
LYSOSOME	FOCAL ADHESION
STARCH AND SUCROSE METABOLISM	B CELL RECEPTOR SIGNALING PATHWAY
ARACHIDONIC ACID METABOLISM	NOD LIKE RECEPTOR SIGNALING PATHWAY
TOLL LIKE RECEPTOR SIGNALING PATHWAY	CELL ADHESION MOLECULES CAMS
HEMATOPOIETIC CELL LINEAGE	TYPE I DIABETES MELLITUS
CYTOKINE CYTOKINE RECEPTOR INTERACTION	HYPERTROPHIC CARDIOMYOPATHY HCM
**AMINO SUGAR AND NUCLEOTIDE SUGAR METABOLISM***	GLUTATHIONE METABOLISM
**PENTOSE PHOSPHATE PATHWAY***	ARGININE AND PROLINE METABOLISM
LEISHMANIA INFECTION	VIRAL MYOCARDITIS
REGULATION OF ACTIN CYTOSKELETON	INSULIN SIGNALING PATHWAY
COMPLEMENT AND COAGULATION CASCADES	MAPK SIGNALING PATHWAY
LEUKOCYTE TRANSENDOTHELIAL MIGRATION	BLADDER CANCER
ETHER LIPID METABOLISM	CHEMOKINE SIGNALING PATHWAY
RIG I LIKE RECEPTOR SIGNALING PATHWAY	INTESTINAL IMMUNE NETWORK FOR IGA PRODUCTION
JAK STAT SIGNALING PATHWAY	NICOTINATE AND NICOTINAMIDE METABOLISM
ECM RECEPTOR INTERACTION	

Enriched in IDH1mut
**HOMOLOGOUS RECOMBINATION***
DNA REPLICATION
**BASE EXCISION REPAIR***
CELL CYCLE
